# Genome-Wide Association Study of Rice Grain Shape and Chalkiness in a Worldwide Collection of Xian Accessions

**DOI:** 10.3390/plants12030419

**Published:** 2023-01-17

**Authors:** Nansheng Wang, Huguang Chen, Yingzhi Qian, Zhaojie Liang, Guiqiang Zheng, Jun Xiang, Ting Feng, Min Li, Wei Zeng, Yaling Bao, Erbao Liu, Chaopu Zhang, Jianlong Xu, Yingyao Shi

**Affiliations:** 1 College of Agronomy, Anhui Agricultural University, Hefei 230036, China; 2 Institute of Crop Sciences, Chinese Academy of Agricultural Sciences, Beijing 100081, China

**Keywords:** rice, GWAS, QTLs, grain shape, chalkiness

## Abstract

Rice (*Oryza sativa* L.) appearance quality, which is mainly defined by grain shape and chalkiness, is an important target in rice breeding. In this study, we first re-sequenced 137 indica accessions and then conducted a genome-wide association study (GWAS) for six agronomic traits with the 2,998,034 derived single nucleotide polymorphisms (SNPs) by using the best linear unbiased prediction (BLUP) values for each trait. The results revealed that 195 SNPs had significant associations with the six agronomic traits. Based on the genome-wide linkage disequilibrium (LD) blocks, candidate genes for the target traits were detected within 100 kb upstream and downstream of the relevant SNP loci. Results indicate that six quantitative trait loci (QTLs) significantly associated with six traits (*qTGW4.1*, *qTGW4.2*, *qGL4.1*, *qGL12.1*, *qGL12.2*, *qGW2.1*, *qGW4.1*, *qGW6.1*, *qGW8.1*, *qGW8.2*, *qGW9.1*, *qGW11.1*, *qGLWR2.1*, *qGLWR2.2*, *qGLWR4.2*, *qPGWC5.1* and *qDEC6.1*) were identified for haplotype analysis. Among these QTLs, two (*qTGW4.2* and *qGW6.1*), were overlapped with *FLO19* and *OsbZIP47*, respectively, and the remaining four were novel QTLs. These candidate genes were further validated by haplotype block construction.

## 1. Introduction

Rice (*Oryza sativa* L.) is a major staple crop in Asian countries, feeding more than half of the world’s population. Higher yield and quality are two main objectives in rice breeding. Although different countries may have different preferences for rice quality, high-quality rice varieties are always favored by both consumers and producers. Rice consumers in India, southern China, Pakistan, Bangladesh, Sri Lanka, some southeast Asian countries and the USA prefer long and thin rice grains with a fluffy to firm texture and medium to high straight-chain starch contents. However, people in Japan, Korea and northern China prefer short, round, soft and sticky rice grains with low straight-chain starch content. The improvement of the quality of major crops through breeding of superior varieties with higher yield, nutrition and resistance is essential for adequate, reliable and sustainable food supply in the world [1,2]. Identification of genes associated with rice grain shape and chalkiness is important for the breeding of modern rice varieties with excellent rice quality.

With the rapid development of economy and the improvement of living standards, rice quality has become a major concern for many rice producers [3,4,5,6,7]. Rice grain quality comprises appearance, cooking, eating, and nutritional and milling quality, among which appearance quality is a key factor affecting its market acceptability [8]. Appearance quality is mainly represented by grain shape and chalkiness. Rice shape is generally described by grain length (GL), grain width (GW), grain thickness (GT) and grain length-to-width ratio (GLWR), and is closely related to grain weight [9,10]. Chalkiness is usually evaluated by the degree of endosperm chalkiness (DEC) and the percentage of grains with chalkiness (PGWC) [11]. Rice varieties with a DEC higher than 25% are generally unacceptable in most world markets. Breeding for rice varieties with desirable appearance is a primary goal of rice breeders, which may be greatly facilitated by a better understanding of the genetic basis for these traits.

Rice grain size and chalkiness are determined by the interaction of genetic and environmental factors [12,13]. Traditional biparental mapping has localized many GL-, GW-, GT- and GLWR-related quantitative trait loci (QTLs) [14,15,16]. For example, to understand the genetic basis of rice appearance quality including grain size and chalkiness, Dai et al. bred a doubled-haploid population of two short-grain hybrid rice varieties, Chunjiang 06 (CJ06) and Rice Taichung Native One (TN1), in subtropical Hangzhou and tropical Hainan. Nineteen main-effect QTLs and nine epistatic interactions controlling grain chalkiness and grain shape were detected [17]. Mei et al. detected 40 QTLs associated with DEC, PGWC and endosperm transparency using three recombinant self-reproducing populations from a three-line cross combination [18]. Moreover, Bian et al. conducted a quantitative genetic analysis of a population consisting of 37 introgression lines (ILs) in two different environments. A total of 54 QTLs were detected on 11 chromosomes, 44 of which were associated with multiple traits [19]. Recently, a genome-wide association study (GWAS) has been employed to detect QTLs associated with quality traits in rice, which is much less time-consuming than traditional techniques and can locate more QTLs or alleles. For example, a previous study detected a total of 16 and 20 QTLs associated with grain appearance quality using single nucleotide polymorphism (SNP) and bin-GWAS methods, and identified dominant alleles for *GS3*, *GW5*, *GL3.1*, *GW7*, *Chalk5* and *qPGWC8.2* [20]. Zhong detected 152, 106 and 12 QTLs for three traits of yield, including GL, GW and GT, in 529 rice varieties using GWAS [21].

The genetic basis for grain shape and chalkiness in rice has been well-studied in the past decades [15,22], and many related genes have been identified and cloned, such as *GW2* [23], *GS3* [24], *GS5* [25], *qGL3* [26], *GW8* [27], *GL7* [28], *GW7* [29], *OsMAPK6* [30], *Chalk5* [31], *TGW6* [32], *GW5* [33], *qSW5* [34]. *GS3* [24] is the major gene controlling GL in rice. Mutation in the second exon will change the cysteine codon (TGC) to a stop codon (TGA) at the protein level, leading to diversity in rice GL. *GW5* [33] is an IQ calmodulin-binding motif family protein that regulates rice GW and thousand grain weight (TGW), and its mutation at the protein level can convert the cysteine codon (TGC) to a stop codon (TGA), resulting in diversity of GL in rice. The seeds of *GW5* loss-of-function mutant were wider than those of the wild type. *Chalk5* [31] is the major QTL regulating seed chalkiness, and also affects rice ears, rice yield and many other quality traits. It is an enzyme encoding a vesicular H++ translocating pyrophosphatase that combines inorganic pyrophosphatase hydrolysis with H+ translocating activity. Plants overexpressing *Chalk5* have higher chalkiness in the endosperm. Although a number of genes controlling quality traits have been identified, further research is needed to elucidate the molecular regulatory mechanisms of rice grain shape, yield and chalkiness.

Although many genes controlling rice grain shape and chalkiness have been cloned and identified, a number of QTLs controlling rice grain shape and grain weight were only detected by initial localization, and many more have not been cloned to date. The isolation of candidate genes by mapping-based cloning is very time-consuming and requires a long time to develop near-isogenic lines for precise localization. GWAS can decipher the relationships between traits and their causal genomic regions. The genetic basis for complex traits can be revealed by studying the diversity of phenotypes and genetic variations in a large number of unrelated relatives [35,36]. In this study, we re-sequenced 137 rice accessions worldwide and obtained approximately 2.89 million SNPs to analyze the genetic basis for rice appearance quality. Six agronomic traits (thousand grain weight (TGW), grain length (GL), grain width (GW), grain length to width ratio (GLWR), percentage of grains with chalkiness (PGWC), and degree of endosperm chalkiness (DEC)) of rice were investigated in three environments. To avoid false positives, we selected the best linear unbiased prediction (BLUP) values for the six agronomic traits within the three environments and conducted genetic diversity analysis for each trait. In addition, we refined the number of candidate genes by combining haplotype block structure analysis with gene function annotation. The results provide an important genomic resource for the molecular breeding of rice and for studying the genetic basis for high yield and quality in rice.

## 2. Results

### 2.1. Phenotypic Variation and Correlation

**Figure 1:**
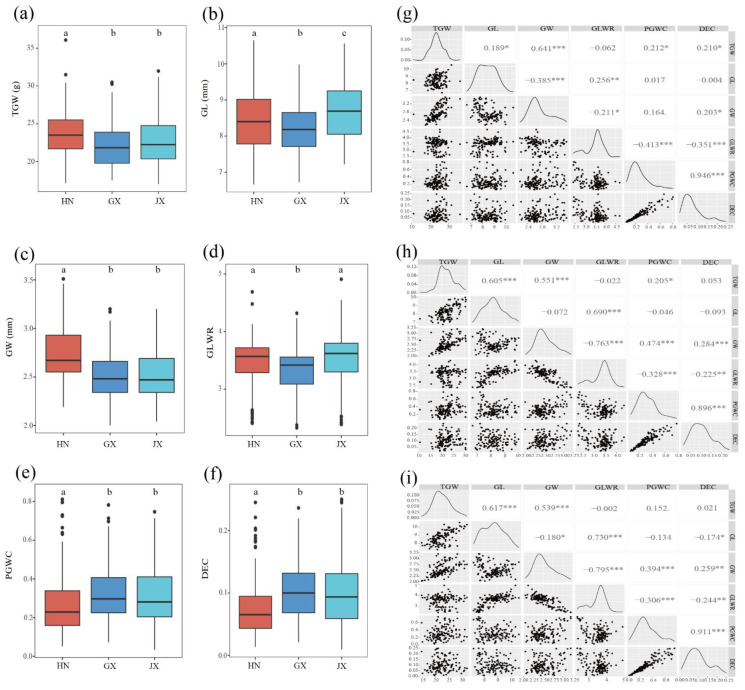
Box plots of six rice grain appearance and milling quality traits in three environments and phenotypic correlations of six traits in different environments. HN, Hainan; GX, Guangxi; JX, Jiangxi; (**a**) thousand grain weight; (**b**) grain length; (**c**) grain width; (**d**) grain length to width ratio; (**e**) percentage of grains with chalkiness; (**f**) degree of endosperm with chalkiness; (**g**) Hainan; (**h**) Guangxi; (**i**) Jiangxi; ‘*’, ‘**’, and ‘***’ refer to significant correlations (*p* < 0.05, *p* < 0.01, and *p* < 0.001).

In general, some traits appeared to be normally distributed, but other traits showed skewed distributions, especially GL, GLWR, PGWC and DEC (Figure S1). The panel showed large variations for all the measured traits. Significant variations between Hainan (HN) and Guangxi (GX) were observed for TGW, GL, GW, GLWR, PGWC and DEC; significant variations in TGW, GL, GW, PGWC and DEC were found between Hainan (HN) and Jiangxi (JX); and significant variations between GX and JX were observed for GL and GLWR (Figure 1a–e).

The TGW value ranged from 17.01 to 36.08 g, with an average value of 23.67, 22.37 and 22.68 g in HN, GX and JX, respectively. GL was both the highest (10.65 mm) and lowest (6.66 mm) in HN, and its average value in the 137 accessions was 8.43, 8.23 and 8.69 mm in HN, GX and JX, respectively. The GW value ranged from 2.00 to 3.51 mm, and was averagely 2.75, 2.32 and 2.52 mm in HN, GX and JX, respectively. The mean of GLWR in the 137 accessions was 3.45, 3.31 and 3.51 in HN, GX and JX, respectively. The GLWR value was the lowest in GX (2.33) and the highest in JX (4.91). PGWC was averagely 0.27, 0.32 and 0.32 in HN, GX and JX, respectively, and ranged from 0.03 to 0.81.

The mean DEC in the 137 accessions was 0.08, 0.10 and 0.10 in HN, GX and JX, respectively. In addition, the minimum DEC value was found in GX and JX (0.01), and the maximum value was observed in GX and JX (0.25). The broad-sense heritability (H_B_^2^) averaged across the three environments was 0.41, 0.52, 0.43, 0.98, 0.78 and 0.67 for TGW, GL, GW, GLWR, PGWC and DEC, respectively (Table 1). ANOVA analysis indicated that the effects of accession, environment and their interaction were highly significant (*p* < 0.001) except for the effect of environment on TGW (*p* = 0.0012) (Table S2). The pairwise correlations between the measured traits were similar in the three environments. TGW was positively correlated with GL, GW, PGWC and DEC, but negatively correlated with GLWR. Positive correlations were observed between DEC, PGWC and GW. GL was negatively correlated with GW. These results demonstrated that rice grain traits are highly related to each other, providing important information for rice grain shape modification (Figure 1g–i).

### 2.2. Phylogenetic and Population Structure Analysis

**Figure 2:**
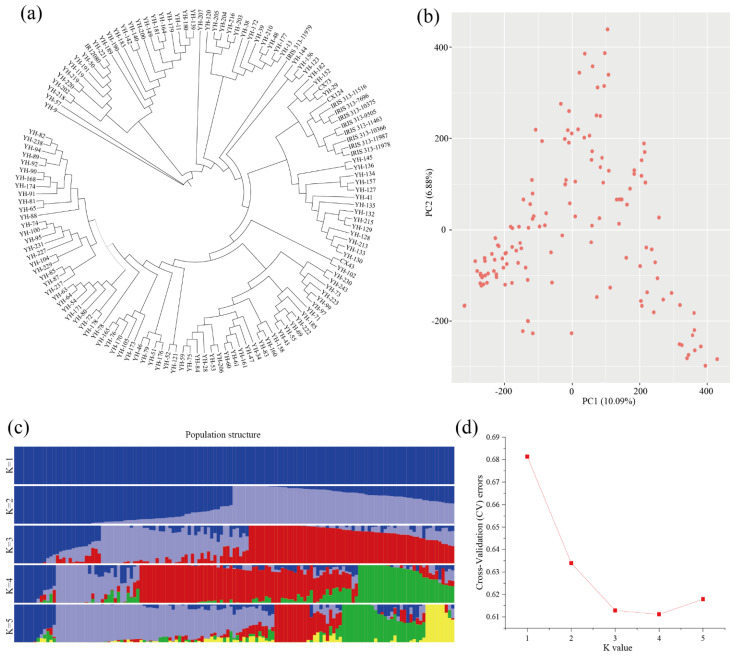
Genetic evolution of natural populations of indica rice. (**a**) Phylogenetic tree; each branch is a rice accession. (**b**) Principal component analysis on 2.89 million SNPs of 137 rice accessions. PC1 and PC2 refer to the first and second principal components, respectively. The numbers in parentheses refer to the proportion of variance explained by the corresponding axes. Red points represent the 137 rice accessions, with each point representing one rice accession. A shorter distance between the points indicates a closer relationship. (**c**) Cluster analysis results of population genotypes, in which each color represents a group and each row indicates a group value. (**d**) Cross validation error rate for each k value. Among them, K is the lowest when k is 4.

The phylogenetic tree (Figure 2a) shows that the structure of the population used in this experiment is uniform without strong population stratification. Based on the SNPs, the Admixture [37] software was used to analyze the population structure. Cross-validation error analysis revealed that the error peak was the lowest at K = 4, indicating that the grouping was optimal (Figure 2c). Population structure analysis showed no obvious pedigree differentiation in the selected plant materials, confirming that they were suitable for subsequent GWAS analysis. The phylogenetic tree shows that the selected population could be divided into four subgroups, which verified the conclusion that K = 4 is the optimal result in the population structure analysis. Therefore, the Q matrix with K = 4 was selected for subsequent association analysis (Figure 2d). Based on the SNP data, R software was used to perform Principal Component Analysis (PCA) [38] to cluster the sample (Figure 2b). The results showed that the 137 materials were not clustered together and were scattered all over the place. The PCA results supported the evolutionary analysis, further confirming that there was a low degree of discreteness of the individual kinship in the population. Kinship, PCA and phylogenetic trees together assess population structure.

### 2.3. Genome-Wide LD Patterns and QTL Detection by GWAS

The maximum LD was 0.30 in the whole population. LD reached half of its initial value at around 92 kb (Figure S2).

**Figure 3:**
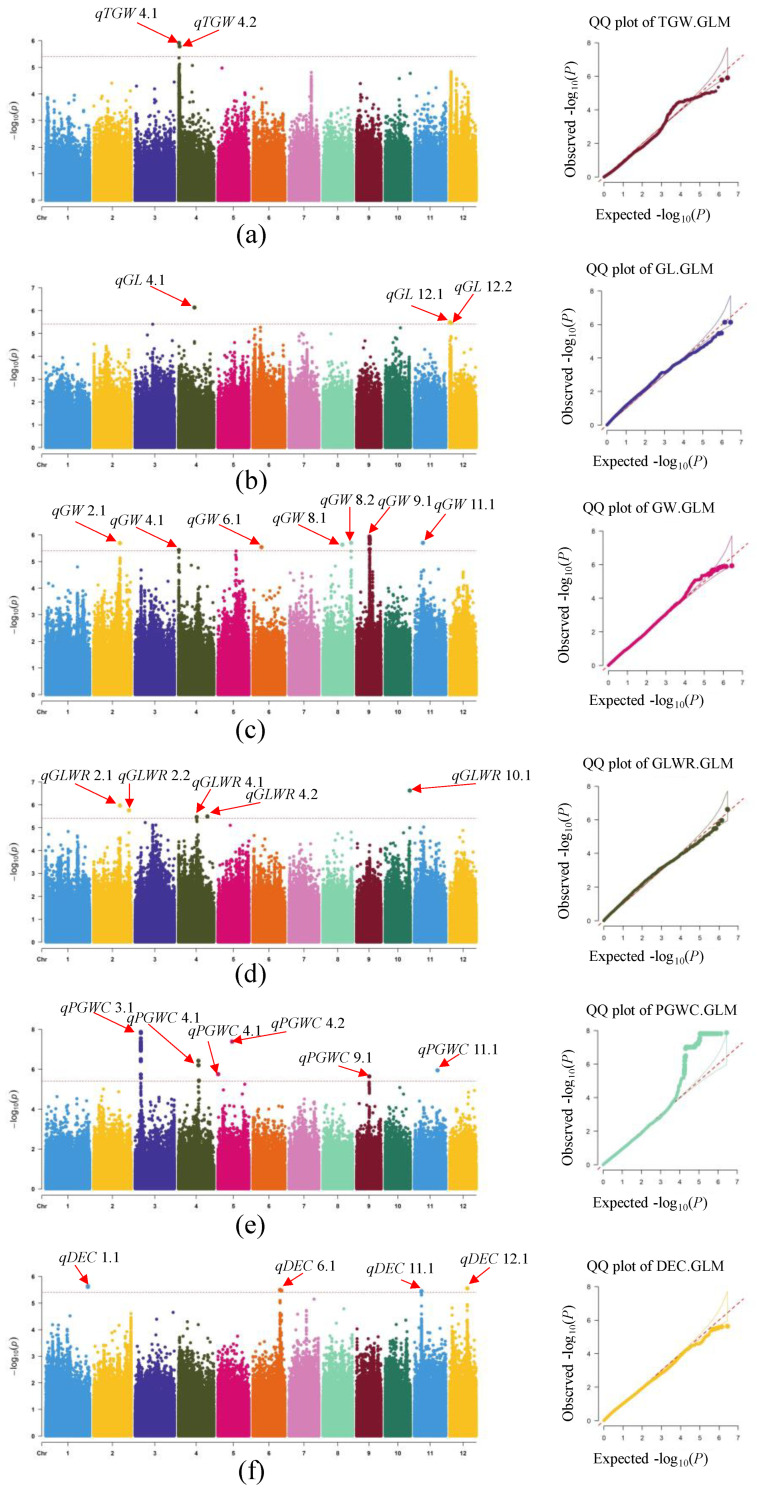
Genome-wide association plots of TGW, GL, GW, GLWR, PGWC and DEC in rice population plotted using the generalized linear model. The Manhattan map of genome-wide scans shows the −log10(*p*) values corresponding to the location of each of 12 chromosomes. Red solid lines represent the whole genome significant threshold *p* = 3.9 × 10^−6^. Red arrows indicate QTLs *qTGW4.1*(rs4_608507), *qTGW4.2*(rs4_1225773), *qGL4.1*(rs4_16205291), *qGL12.1*(rs12_225117), *qGL12.2*(rs12_1188996), *qGW2.1*(rs2_26502715), *qGW4.1*(rs4_502118), *qGW6.1*(rs6_8695272), *qGW8.1*(rs8_19551373), *qGW8.2*(rs8_28472120), *qGW9.1*(rs9_13050866), *qGW11.1*(rs11_8695392), *qGLWR2.1*(rs2_26502715), *qGLWR2.2*(rs2_35438743), *qGLWR4.1*(rs4_18391272), *qGLWR4.2*(rs4_29199879), *qGLWR10.1*(rs10_24820313), *qPGWC3.1*(rs3_5812574), *qPGWC4.1*(rs4_20153796), *qPGWC5.1*(rs5_267911), *qPGWC5.2*(rs5_14313526), *qPGWC9.1*(rs9_12763755), *qPGWC11.1*(rs11_23382869), *qDEC1.1*(rs1_42431688), *qDEC6.1*(rs6_27376022), *qDEC11.1*(rs11_7169791) and *qDEC12.1*(rs12_17698258) colocalized by GLM. The horizontal axis in the quantile–quantile (QQ) plot represents the expected value of the −log10 transformation, whereas the vertical axis indicates the observed value of the −log10 transformation. Manhattan plot and QQ plot of TGW (**a**), GL (**b**), GW (**c**), GLWR (**d**), PGWC (**e**) and DEC (**f**) in GLM.

In this study, the association analysis was performed using the BLUP method for each accession to reduce the environment effects and simplify the calculation. The general linear model (GLM) was used to conduct GWAS on grain weight, grain shape and chalkiness (TGW, GL, GW, GLWR, PGWC and DEC). Considering the decay distance of LD in rice, adjacent SNPs with spans less than 200 kb were defined as single QTLs, and the SNP with the lowest *p* value was taken as the lead SNP to reduce redundant association signals of different traits. Stringent criterion of −log10 (*p*) > 5.4 in the three environments was used to determine the association significance of grain weight, grain shape and chalkiness (Table S3). The results showed that 195 SNPs had significant associations with six agronomic traits. We successfully identified both known genes and previously reported QTLs from rice as well as some novel candidate loci in rice genome. The results revealed that two, three, seven, five, six and four QTLs detected by GLM were significantly correlated with TGW, GL, GW, GLWR, PGWC and DEC, respectively (Table 2). A total of 379 candidate genes were obtained (Table S4). In general, two QTL regions (*qTGW4.1* and *qTGW4.2* on chromosome 4) were determined to be significantly correlated with TGW, which accounted for 17.7% and 20.4% of the phenotypic variance, respectively (Table 2, Figure 3a). The three QTLs associated with GL were *qGL4.1* on chromosome 4 and *qGL12.1* and *qGL12.2* on chromosome 12, which accounted for 18.0%, 15.7% and 15.7% of the phenotypic variance, respectively (Table 2, Figure 3b). Seven QTLs were significantly correlated with GW, including *qGW2.1* on chromosome 2, *qGW4.1* on chromosome 4, *qGW6.1* on chromosome 6, *qGW8.1* and *qGW8.2* on chromosome 8, *qGW9.1* on chromosome 9 and *qGW11.1* on chromosome 11. These QTLs explained 13.9–20.2% of the total phenotypic variance. (Table 2, Figure 3c). Five QTLs were significantly associated with GLWR, including *qGLWR2.1* and *qGLWR2.2* on chromosome 2, *qGLWR4.1* and *qGLWR4.2* on chromosome 4 and *qGLWR10.1* on chromosome 10, which accounted for 14.0–19.3% of the phenotypic variance (Table 2, Figure 3d). Six QTLs were significantly correlated with PGWC, including *qPGWC3.1* on chromosome 3, *qPGWC4.1* on chromosome 4, *qPGWC5.1* and *qPGWC5.2* on chromosome 5, *qPGWC9.1* on chromosome 9 and *qPGWC11.1* on chromosome 11, which accounted for 21.8%, 20.1%, 19.8%, 20.3%, 21.1% and 18.7% of the phenotypic variance, respectively (Table 2, Figure 3e). Four QTLs had significant correlations with DEC, including *qDEC1.1* on chromosome 1, *qDEC6.1* on chromosome 6, *qDEC11.1* on chromosome 11 and *qDEC12.1* on chromosome 12. They accounted for 16.3%, 17.0%, 17.3% and 16.6% of the total phenotypic variance, respectively (Table 2, Figure 3f).

### 2.4. Candidate Gene Identification and Haplotype Analysis

**Figure 4:**
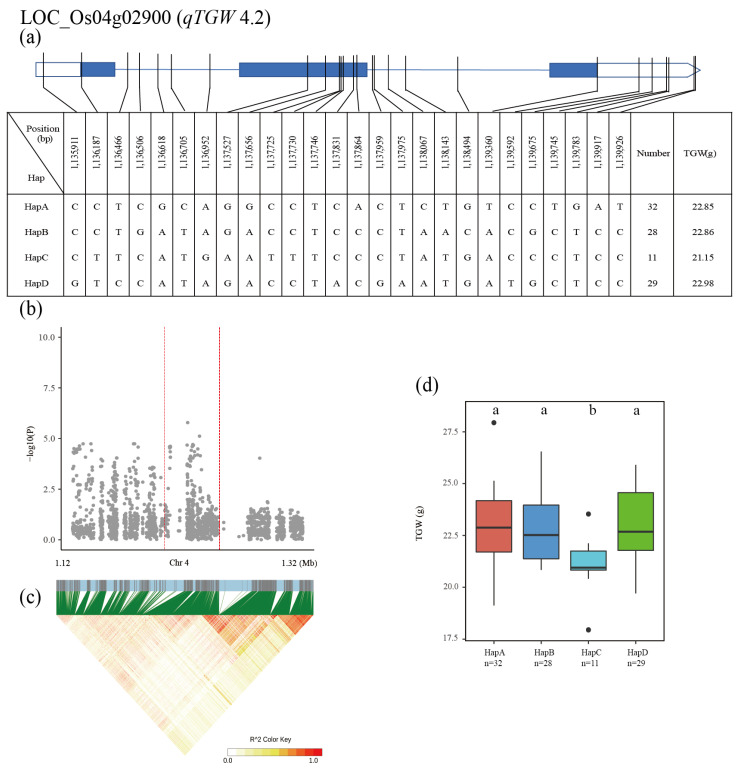
Identification of candidate genes for TGW. (**a**) Based on 26 SNPs in all evaluated rice accessions, four haplotypes of LOC_Os04g02900 were identified. In the gene structure diagram of LOC_Os04g02900 (http://rice.plantbiology.msu.edu, accessed on 15 May 2022), the promoter is indicated by white frame; the exon is represented by blue frame; and the intron and intergenic region are marked by blue lines. A thin black line represents the genomic location of each SNP. Haplotypes with fewer than 10 accessions are not shown. TGW (**b**) based on single polymorphism and LD heat map of local Manhattan map (**c**), around the peak on chromosome 4. Red dotted lines represent candidate regions for associated SNPs. Based on TGW (**d**) of LOC_Os04g02900 haplotype, differences between haplotypes were statistically analyzed using Tukey’s test.

The twenty-seven identified QTLs were used for high-density association and gene-based haplotype analysis to identify the candidate genes. In the region of *qTGW4.2* (1.12–1.32 Mb on chromosome 4), the 2795 SNPs of ten genes were used for high-density association analysis. The annotated gene with the most significant hit was LOC_Os04g02900 (Figure 4b,c). Four major haplotypes were detected among the 137 accessions based on eight SNPs in the LOC_Os04g02900 promoter, eight SNPs in the exon, and ten SNPs in the intron. The mean TGW was 22.85, 22.86, 21.15 and 22.98 g for HapA, HapB, HapC and HapD, respectively (Figure 4a). Haplotype analysis of the whole population revealed that HapD had a significantly higher TGW than other three haplotypes. Significant differences in TGW were observed among the four haplotypes in the population (Figure 4d).

**Figure 5:**
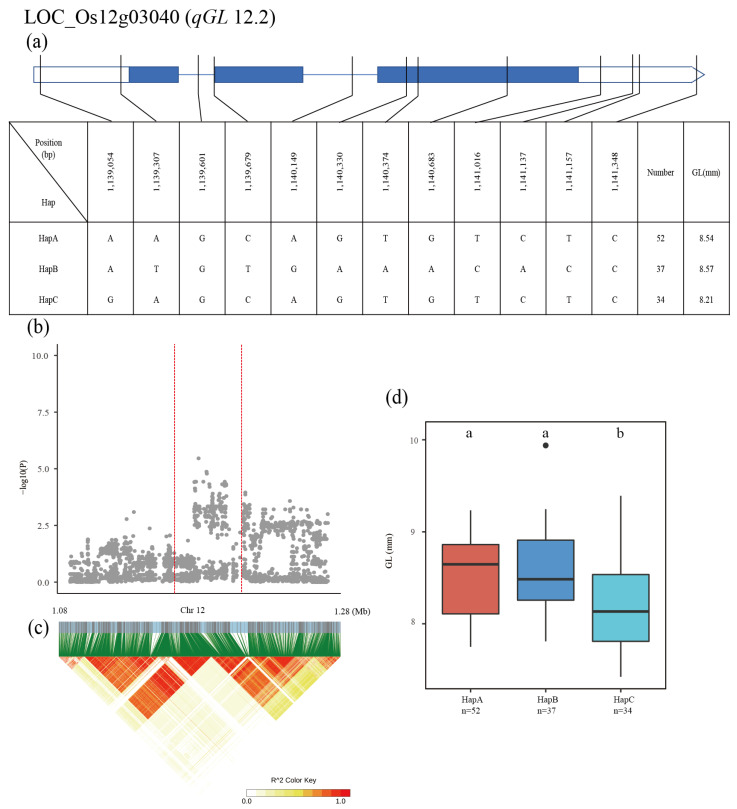
Identification of candidate genes for GL. (**a**) Based on 12 SNPs in all evaluated rice accessions, three haplotypes of LOC_Os12g03040 were identified. In the gene structure diagram of LOC_Os12g03040, the promoter is indicated by white frame; the exon is represented by blue frame; and the intron and intergenic region are marked by blue lines. A thin black line represents the genomic location of each SNP. Haplotypes with fewer than 10 accessions are not shown. GL (**b**) based on single polymorphism and LD heat map of local Manhattan map (**c**), around the peak on chromosome 12. Red dotted lines represent candidate regions for associated SNPs. Based on GL (**d**) of LOC_Os12g03040 haplotype, differences between haplotypes were statistically analyzed using Tukey’s test.

The QTL *qGL12.2* was identified in a 1.08–1.28 Mb region on chromosome 12, and the 1660 SNPs of 21 genes were used for high-density association analysis. The most significant hit was located in LOC_Os12g03040 (Figure 5b,c). Three major haplotypes were detected among the 137 accessions based on six SNPs in the LOC_Os12g03040 promoter, four SNPs in the exon, and two SNPs in the intron. The mean GL was 8.54, 8.57 and 8.21 mm for HapA, HapB and HapC, respectively (Figure 5a). HapB had a significantly higher GL than the other two haplotypes, and there was a significant difference between HapA and HapC in GL (Figure 5d).

**Figure 6:**
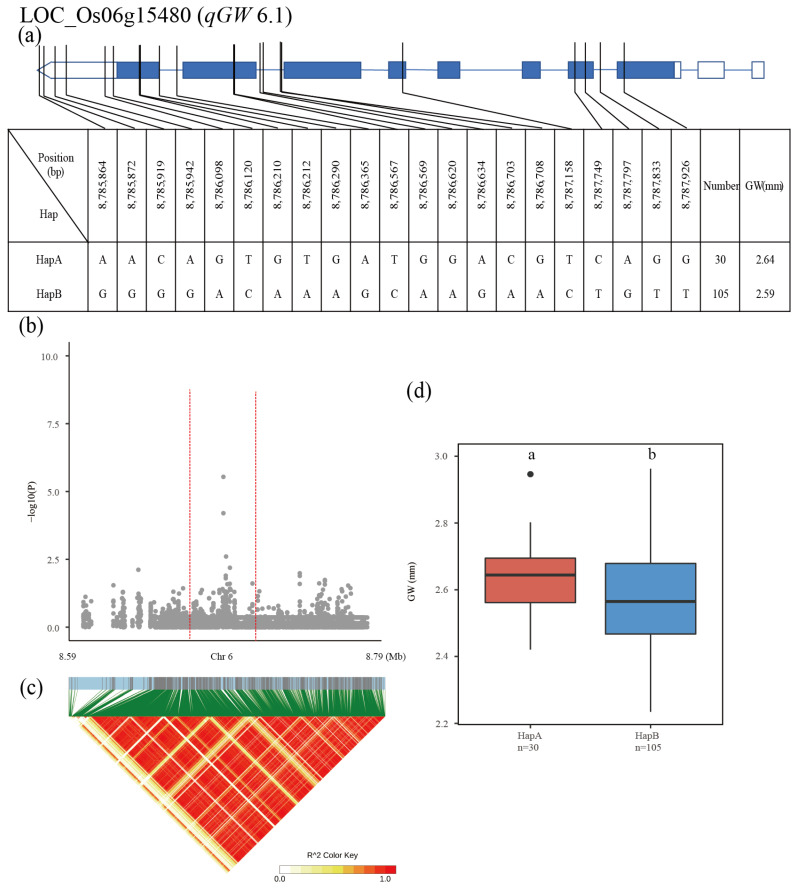
Identification of candidate genes for GW. (**a**) Based on 21 SNPs in all evaluated rice accessions, two haplotypes of LOC_Os06g15480 were identified. In the gene structure diagram of LOC_Os06g15480, the promoter is indicated by white frame; the exon is represented by blue frame; and the intron and intergenic region are marked by blue lines. A thin black line represents the genomic location of each SNP. Haplotypes with fewer than 10 accessions are not shown. GW (**b**) based on single polymorphism and LD heat map of local Manhattan map (**c**), around the peak on chromosome 6. Red dotted lines represent candidate regions for associated SNPs. Based on GW (**d**) of LOC_Os06g15480 haplotype, differences between haplotypes were statistically analyzed using Tukey’s test.

The QTL *qGW6.1* was detected in the region from 8.59 Mb to 8.79 Mb on chromosome 6, and the 2313 SNPs of ten genes were used for association analysis. LOC_Os06g15480 was subsequently screened as the candidate gene for *qGW6.1* (Figure 6b,c). Two major haplotypes were detected among the 137 accessions based on six SNPs in the LOC_Os06g15480 promoter, nine SNPs in the exon and six SNPs in the intron. The average GW was 2.64 mm for HapA and 2.59 mm for HapB, showing significant differences from each other (Figure 6a,d).

**Figure 7:**
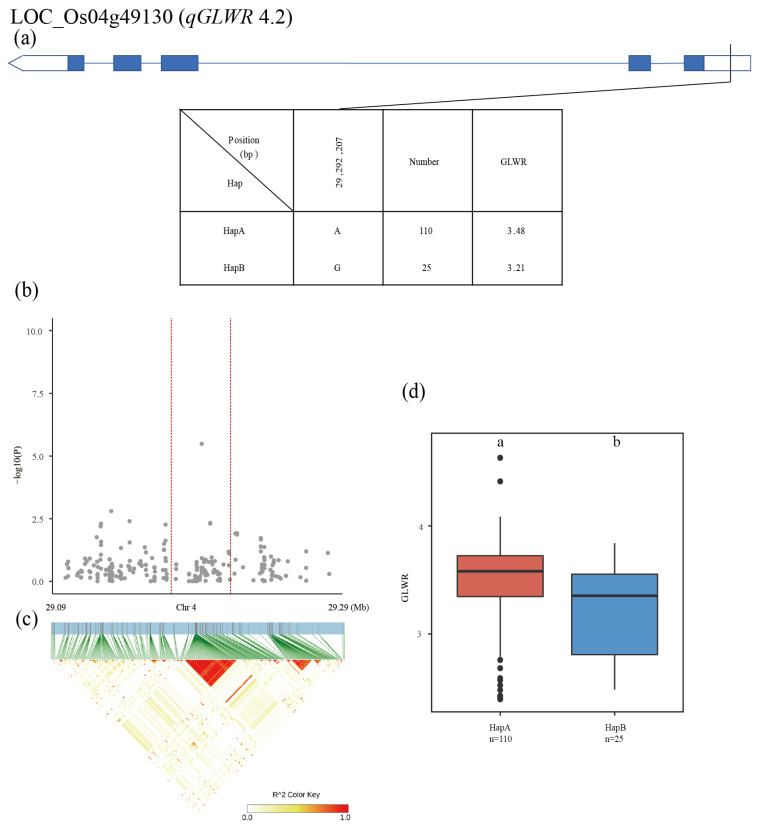
Identification of candidate genes for GLWR. (**a**) Based on one SNP in all evaluated rice accessions, two haplotypes of LOC_Os04g49130 were identified. In the gene structure diagram of LOC_Os04g49130, the promoter is indicated by white frame; the exon is represented by blue frame; and the intron and intergenic region are marked by blue lines. A thin black line represents the genomic location of each SNP. Haplotypes with fewer than 10 accessions are not shown. GLWR (**b**) based on single polymorphism and LD heat map of local Manhattan map (**c**), around the peak on chromosome 4. Red dotted lines represent candidate regions for associated SNPs. Based on GLWR (**d**) of LOC_Os04g49130 haplotype, differences between haplotypes were statistically analyzed using Tukey’s test.

The QTL *qGLWR4.2* was identified in a 29.0–29.2Mb region on chromosome 4, and the 345 SNPs of 15 genes were used for high-density association analysis. The most significant hit was located in LOC_Os04g49130 (Figure 7b,c). Two major haplotypes were detected among the 137 accessions based on one SNP in the LOC_Os04g49130 promoter. The average GLWR was 3.48 for HapA and 3.21 for HapB with significant differences from each other (Figure 7a,d).

**Figure 8:**
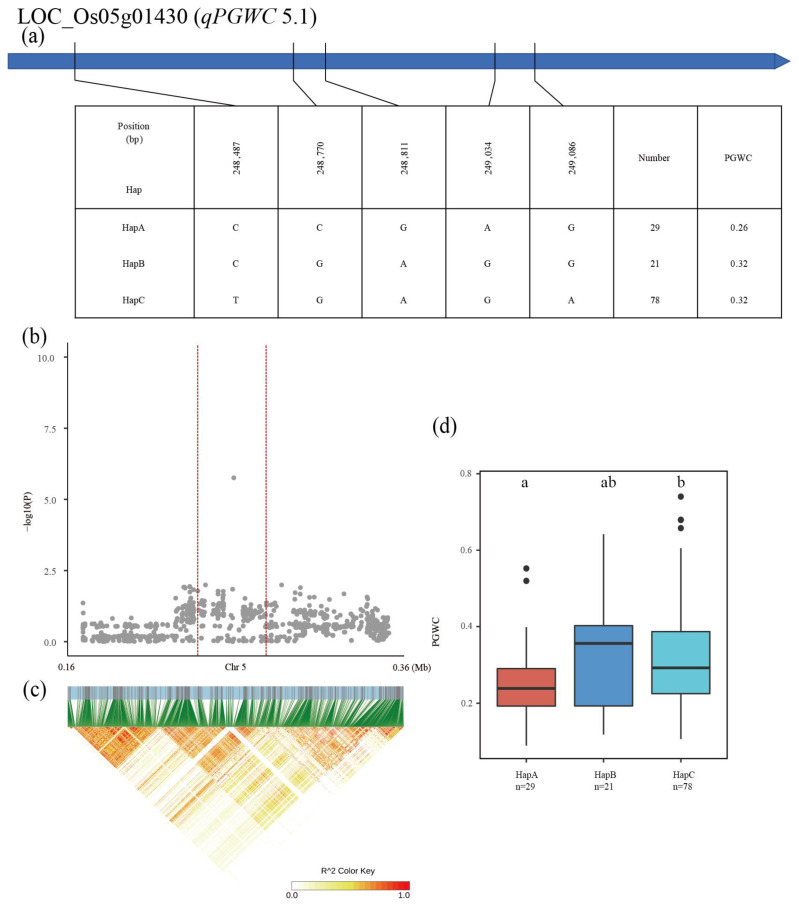
Identification of candidate genes for PGWC. (**a**) Based on five SNPs in all evaluated rice accessions, three haplotypes of LOC_Os05g01430 were identified. In the gene structure diagram of LOC_Os05g01430, the exon is represented by blue frame. A thin black line represents the genomic location of each SNP. Haplotypes with fewer than 10 accessions are not shown. PGWC (**b**) based on single polymorphism and LD heat map of local Manhattan map (**c**), around the peak on chromosome 5. Red dotted lines represent candidate regions for associated SNPs. Based on PGWC (**d**) of LOC_Os05g01430 haplotype, differences between haplotypes were statistically analyzed using Tukey’s test.

The QTL *qPGWC5.1* was detected in the region from 0.16 Mb to 0.36 Mb on chromosome 5, and the 952 SNPs of 28 genes were used for the association analysis. LOC_Os05g01430 was subsequently screened as the candidate gene for *qPGWC5.1* (Figure 8b,c). Three major haplotypes were detected among the 137 accessions based on five SNPs in the LOC_Os05g01430 exon. The mean PGWC was 0.26, 0.32 and 0.32 for HapA, HapB, and HapC, respectively (Figure 8a). The PGWC of HapA was significantly lower than that of other two haplotypes (Figure 8d).

**Figure 9:**
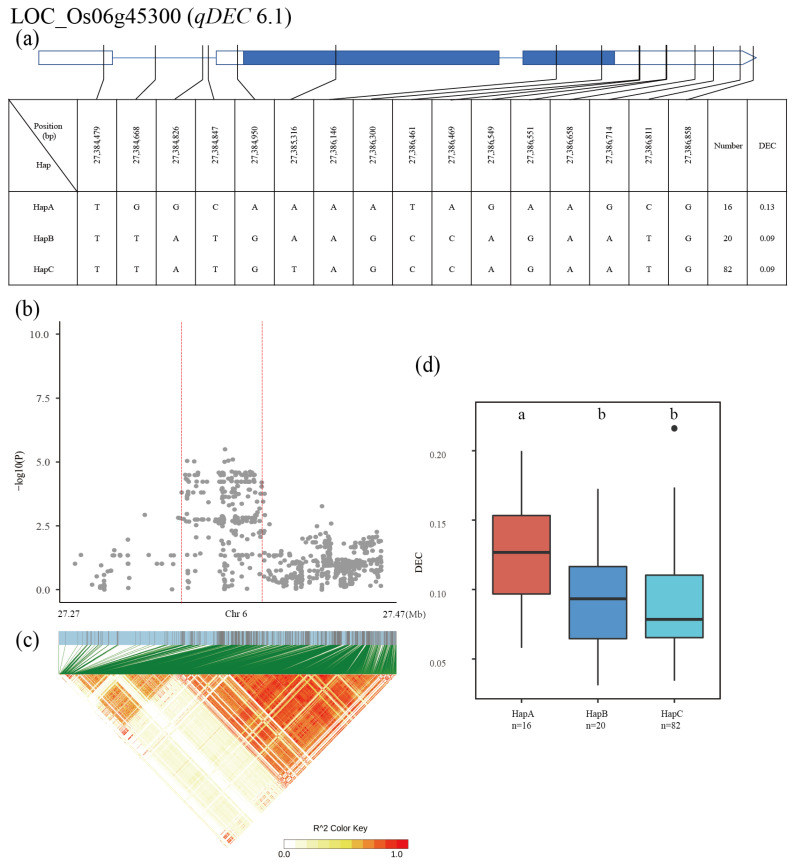
Identification of candidate genes for DEC. (**a**) Based on 16 SNPs in all evaluated rice accessions, three haplotypes of LOC_Os06g45300 were identified. In the gene structure diagram of LOC_Os06g45300, the promoter is indicated by white frame; the exon is represented by blue frame; and the intron and intergenic region are marked by blue lines. A thin black line represents the genomic location of each SNP. Haplotypes with fewer than 10 accessions are not shown. DEC (**b**) based on single polymorphism and LD heat map of local Manhattan map (**c**), around the peak on chromosome 6. Red dotted lines represent candidate regions for associated SNPs. Based on DEC (**d**) of LOC_Os06g45300 haplotype, differences between haplotypes were statistically analyzed using Tukey’s test.

The QTL *qDEC6.1* was identified in a 27.27–27.47 Mb region on chromosome 6, and the 1056 SNPs of 28 genes were used for high-density association analysis. The most significant hit was located in LOC_Os06g45300 (Figure 9b,c). Three major haplotypes were detected among the 137 accessions based on ten SNPs in the LOC_Os06g45300 promoter, three SNPs in the exon, and three SNPs in the intron. The mean DEC was 0.13, 0.09 and 0.09 for HapA, HapB and HapC, respectively (Figure 9a). The DEC of HapA was significantly higher than that of other two haplotypes (Figure 9d).

## 3. Discussion

The availability of high-density genotype data provided by next generation sequencing offers great opportunities to re-analyze previously collected panels of phenotypes. High-density SNP datasets can provide higher genomic coverage and resolution, and GWAS can identify segregating loci in populations [41], loci with reduced genetic background and QTL–environment interactions [42,43,44]. The use of larger structured populations may improve the mapping resolution for detecting global QTLs. Regression models are often constructed to test the correlations between markers and phenotypes. Population structure is usually represented by the proportion of subpopulations to which individuals belong, which is also known as the Q-matrix. Since the subsets in the Q matrix have the fitting of fixed effects, the general linear model (GLM) can be used to test for genetic markers [38,45]. This model can be conceptually expressed as y = Q + S + e, where y and e are the phenotype and residue, respectively.

In this study, we genotyped 137 indica rice accessions using 2,998,034 SNPs (Figure S3). We found sufficient diversity to map QTLs associated with rice grain weight, grain size and chalkiness, and identified haplotypes with significant differences in grain weight, grain size and chalkiness. Although the natural population consisting of 137 accessions was not large enough, there were significant phenotypic variations in grain weight, grain shape and chalkiness. In this study, the variation coefficients of TGW, GL, GW, GLWR, PGWC and DEC were 13.62–14.69%, 8.77–9.10%, 9.85–11.37%, 12.65–13.65%, 43.07–59.11% and 44.21–62.36% in the whole population, respectively. Particularly, HN had the highest TGW (36.08 g), GL (10.65 mm), GW (3.51 mm) and PGWC (0.81). These significant phenotypic variations may be associated with high genetic diversity.

By using GWAS and gene-based association analysis, combined with haplotype analysis of candidate genes, we screened six candidate genes for six important QTLs controlling the measured traits. Of the three major components (panicle number per plant, number of grains per panicle and grain weight) of rice yield, grain weight measured as TGW is the most stable and heritable trait. In this study, one promising gene located in the QTL for grain weight was also identified. For *qTGW4.2*, LOC_Os04g02900 (*FLO19*) [39] encodes a pyruvate dehydrogenase complex E1-alpha subunit involved in the biosynthesis of galactolipids, which is required for the development of amyloplasts. The mutation of this gene significantly decreased the pyruvate dehydrogenase complex enzyme activity, accompanied by a significant decrease in total galactolipid content, which led to the abnormal development of amyloplasts, impaired starch synthesis and ultimately seriously affected rice yield and quality.

Rice grains grow inside the spikelet hull with a limited caryopsis space. Therefore, rice grain shape and size are strictly determined by the maternal genotype that controls the cell number and size of glumes. The genes cloned so far provide insights into the regulatory pathway of grain shape and size, including the ubiquitin-proteasome, G-protein signaling, and mitogen-activated protein kinase (MAPK) signaling pathways, as well as plant hormones and transcriptional regulators [46,47]. In the present study, three promising genes (one known gene and two novel genes) located in the three new QTLs for grain weight were identified using a large natural population with 137 accessions. The first one was *qGL12.2*, in which LOC_Os12g03040 is annotated as a NAC (NAM/ATAF/CUC) transcription factor playing important roles in regulating plant growth and development as well as biotic and abiotic stress responses. The second one was *qGW6.1*, in which LOC_Os06g15480 is annotated as a basic region-leucine zipper (bZIP) transcription factor. This gene has been identified in a previous study and designated as *OsbZIP47* [40] with transcriptional activation activity, but wg1 can directly interact with *OsbZIP47* and recruit the transcriptional co-repressor aberrant spikelet and panicle1 (*ASP1*) to repress its transcriptional activity. *OsbZIP47* overexpression lines showed narrower seeds, which is similar to the *wg1* mutant. The *wg1-1 osbzip47-c2* double mutant had wider and heavier seeds than the *wg1-1* single mutant, and *OsbZIP47* was found to negatively regulate grain width by limiting cell proliferation. The third one was *qGLWR4.2*, in which LOC_Os04g49130 is annotated as a small ubiquitin-like modifier (SUMO)-conjugating enzyme E2. SUMOylation modification is an important eukaryotic post-translational modification regulating many cellular processes in plants, from seed development to stress response. The transcript levels of SUMOylation target genes, including Abscisic Acid (ABA) and gibberellins (GA) associated with cystatin-related epididymal spermatogenic (CREs), are responsive to treatment with these hormones.

Chalkiness is caused by the deposition of starch and storage proteins in the endosperm, which is tightly related to grain filling. Grain filling is a dynamic process related to the source–sink balance and controlled by a complex genetic mechanism and sensitive to environmental conditions [48,49,50]. In this study, two promising genes located in the two new QTLs for grain weight were identified using a large natural population with 137 accessions. The first one was *qPGWC5.1*, from which LOC_Os05g01430 was screened as a candidate gene and annotated as polygalacturonase inhibiting protein 3. LOC_Os05g01430 is a plant cell wall glycoprotein inhibiting fungal endopolygalacturonases and modulating their activity to promote the accumulation of elicitor-active oligogalacturonides. The second one was *qDEC6.1*, in which LOC_Os06g45300 was identified as the candidate gene and annotated as a rice dual-specificity protein kinase. Dual-specificity protein kinases are a group of protein kinases able to phosphorylate both tyrosine and serine/threonine residues. These results improve our understanding of the genetic basis for grain weight, grain shape and chalkiness in rice and provide valuable information for elucidating the molecular mechanisms underlying these traits.

Overall, we obtained a set of QTLs significantly associated with grain weight, grain shape and chalkiness in rice through the GWAS analysis of 137 rice accessions. Candidate genes significantly associated with agronomic traits and functional annotation of each gene were further screened by haplotype block structure analysis. As expected, although a large LD contains many SNPs in a candidate region, our results suggest that the number of candidate genes can be significantly reduced by combining haplotype block structure analysis and gene function annotation. In conclusion, our findings will contribute to future gene functional analysis and provide valuable information for rice gene cloning.

## 4. Materials and Methods

### 4.1. Plant Materials, Field Trials and Trait Measurements

A total of 137 accessions (Table S1) from the world were used to test the association between the SNP genotype and the phenotype of grain weight, grain shape and chalkiness. All of these accessions were grown in three environments, including Hainan (18.3 N, 109.3 E; HN), Guangxi (20.54 N, 104.29 E; GX) and Jiangxi (24.29 N, 118.28 E; JX) in 2018. In all the three environments, each accession was planted in a two-row plot with 10 individuals in each row at a spacing of 20 cm × 25 cm with two replicates for each accession. Field management included irrigation, fertilizer application and pest control, following normal agricultural practices. At maturity (about 40 days after flowering), seeds of ten plants in the middle of each plot were harvested, air-dried and stored at room temperature for at least three months before testing [51]. Then, all full head milled rice kernels of each accession were used to measure the grain length (GL, mm), grain width (GW, mm), grain length-width ratio (GLWR), degree of endosperm chalkiness (DEC) and percentage of grains with chalkiness (PGWC) using a rice grain appearance quality scanning machine ((Model SC-G, Hangzhou, China, http://www.wseen.com/, accessed on 20 April 2022). Subsequently, the weight of these seeds was measured using a high precision electronic balance (1/1000, APTP456 series) and the TGW in grams was subsequently calculated. The scanner was calibrated with a calibration target before each measurement.

### 4.2. Statistical Analysis

Excel 2018 was employed for data compilation, and the mean, standard deviation and coefficient of variation of each trait were calculated. The correlation analysis and frequency analysis of six grain quality-related traits were carried out by R (TGW, GL, GW, GLWR, PGWC and DEC). The R package ‘lme4’ [52] was used to obtain the best linear unbiased estimate (BLUP) for each genotype–environment combination and variance components using generalized linear models.

### 4.3. DNA Extraction and SNP Genotyping

For each of the 137 accessions to be sequenced, two leaves were collected from a single plant at the tillering stage (one month after seedling transplantation), and genomic DNA was extracted using a standard cetyltrimethylammonium bromide protocol [53]. According to the manufacturer’s instructions (Illumina, https://www.illlighta.com/, accessed on 26 April 2019), paired-end sequencing libraries were constructed using 5 µg of genomic DNA, with inserted fragments of approximately 350 bp. The Illumina HiSeq X10 platform was used to obtain the pair-ends of 150 bp reads, and the original sequence was further processed to remove adaptor-containing and low-quality reads. Library construction, sequencing and sequence cleaning were all performed by BGI Shenzhen Company. The reference genome was Shuhui 498 (R498). GATK was used to call SNPs [54]. The mapping results were converted to VCF format using SAMtools (version 0.1.18) [55]. SNPs with MAF ≥ 5% and missing rate ≤ 20% were retained. IMPUTE2 [56] was used to impute missing genotypes, and 2,898,034 high-quality SNPs were finally obtained.

### 4.4. Population Structure and Kinship Analysis

The TASSEL [57] was used to calculate the population structure (Q) and kinship (K). All SNPs were used in the calculation. Principal component analysis (PCA) was used to evaluate the population structure. The PCA score and relationship matrix would be used in the generalized linear model (GLM) [38] below.

SNP data of candidate genes were extracted based on genotype of SNP with MAF > 0.05. This dataset only contained double-allele SNPs. Further haplotype analysis excluded heterozygotes and missing alleles. Haplogroups consisting of fewer than 10 accessions were deleted. For the genes found in QTLs, haplotype analysis only used the non-synonymous SNPs in the coding region of these genes for haplotype analysis of R.

### 4.5. Linkage Disequilibrium Analysis

The software “PopLDdecay” was used to calculate the linkage disequilibrium (LD) between pairs of markers [58]. “R2” was the square of the Pearson’s correlation coefficient. When the correlation coefficient (R2) dropped to half of its maximum value, the distance across the chromosome is called the LD decay distance [59].

### 4.6. Genome-Wide Association Mapping

In this study, we obtained 2,898,034 SNPs (MAF > 0.05) and six sets of phenotypic data. The SNPs and phenotypic data were used to conduct GWAS in the TASSEL (version 5.2.40) [57] software using a GLM. For the GLM, the suggested *p* value of dominance was 3.9 × 10^−6^ to control the genetic false positive error rate of the population. The SNPs in the same LD region were regarded as one QTL. Here, by referring to the previous report, we used the LD decay distance of 100 kb [60], and the SNP with the lowest *p*-value was regarded as the lead SNP. The Manhattan plot was drawn using the R package “CMplot”.

### 4.7. Identification of Candidate Genes and Haplotype Analysis

In order to identify candidate genes related to TGW, GL, GW, GLWR, PGWC and DEC, the rice genome annotation project (http://rice.plantbiology.msu.edu, accessed on 26 April 2022) was used to search for candidate genes in the 200 kb genomic region of the selected SNPs. Among all candidate genes, four types of genes, including expression proteins, hypothetical proteins, retrotransposons and transposons, were excluded. The SNP data of candidate genes were extracted based on genotype of SNP with MAF > 0.05. This dataset only contained double-allele SNPs. Further haplotype analysis excluded heterozygotes and missing alleles. Haplogroups consisting of fewer than 10 accessions were deleted. For the genes found in QTLs, only non-synonymous SNPs in the coding region of these genes were used for the haplotype analysis of R, and Student’s t-test was performed to determine whether this locus could cause changes in rice rain weight, grain shape and chalkiness. R was used to visualize the results.

## 5. Conclusions

There are considerable genetic variations for six grain quality traits in the panel consisting of 137 rice accessions. Twenty-seven QTLs were identified using GWAS. Six candidate genes were also screened by high-density association and gene-based haplotype analysis. The findings improve our understanding of the genetic basis for grain weight, grain shape and chalkiness in rice and provide valuable information for elucidating the molecular mechanisms underlying these traits. This study also provides a reference for future marker-assisted breeding with QTL or gene pyramiding to stabilize and improve rice quality.

## Figures and Tables

**Table 1:** Statistics of TGW, GL, GW, GLWR, PGWC and DEC in different environments.

Phenotype	Env.	Mean ± SD	Max	Min	CV (%)	H_B_^2^
TGW (g)	HN	23.67 ± 3.22	36.08	17.15	13.62%	0.41
	GX	22.37 ± 3.16	30.50	17.53	14.13%	
	JX	22.68 ± 3.33	31.98	17.01	14.69%	
GL (mm)	HN	8.43 ± 0.77	10.65	6.66	9.10%	0.52
	GX	8.23 ± 0.72	9.98	6.72	8.77%	
	JX	8.69 ± 0.77	10.56	7.22	8.90%	
GW (mm)	HN	2.75 ± 0.31	3.51	2.19	11.37%	0.43
	GX	2.52 ± 0.25	3.20	2.00	9.85%	
	JX	2.52 ± 0.25	3.20	2.04	10.10%	
GLWR	HN	3.45 ± 0.45	4.69	2.41	13.15%	0.98
	GX	3.31 ± 0.42	4.32	2.33	12.65%	
	JX	3.51 ± 0.48	4.91	2.39	13.65%	
PGWC	HN	0.27 ± 0.16	0.81	0.05	59.11%	0.78
	GX	0.32 ± 0.14	0.78	0.07	43.07%	
	JX	0.32 ± 0.16	0.75	0.03	51.09%	
DEC	HN	0.08 ± 0.05	0.25	0.01	62.36%	0.67
	GX	0.10 ± 0.05	0.24	0.02	44.21%	
	JX	0.10 ± 0.06	0.25	0.01	55.31%	

**Table 2:** Twenty-seven QTLs with significant associations with TGW, GL, GW, GLWR, PGWC and DEC.

Trait	QTL	Chr	Lead SNP (bp)	R^2^ (%)	*p* Value	Known Genes/QTLs
TGW	*qTGW4.1*	4	608,507	17.7%	1.22 × 10^−6^	
	*qTGW4.2*	4	1,225,773	20.4%	1.64 × 10^−6^	*FLO19* [39]
GL	*qGL4.1*	4	16,205,291	18.0%	7.28 × 10^−7^	
	*qGL12.1*	12	225,117	15.7%	3.24 × 10^−6^	
	*qGL12.2*	12	1,188,996	15.7%	3.47 × 10^−6^	
GW	*qGW2.1*	2	26,502,715	16.4%	2.03 × 10^−6^	
	*qGW4.1*	4	502,118	15.7%	3.70 × 10^−6^	
	*qGW6.1*	6	8,695,272	13.9%	2.88 × 10^−6^	*OsbZIP47* [40]
	*qGW8.1*	8	19,551,373	20.2%	2.32 × 10^−6^	
	*qGW8.2*	8	28,472,120	17.8%	1.98 × 10^−6^	
	*qGW9.1*	9	13,050,866	17.1%	1.15 × 10^−6^	
	*qGW11.1*	11	8,695,392	16.8%	1.99 × 10^−6^	
GLWR	*qGLWR2.1*	2	26,502,715	17.4%	1.07 × 10^−6^	
	*qGLWR2.2*	2	35,438,743	17.5%	1.73 × 10^−6^	
	*qGLWR4.1*	4	18,391,272	16.1%	3.43 × 10^−6^	
	*qGLWR4.2*	4	29,199,879	14.0%	3.23 × 10^−6^	
	*qGLWR10.1*	10	24,820,313	19.3%	2.36 × 10^−7^	
PGWC	*qPGWC3.1*	3	5,812,574	21.8%	1.35 × 10^−8^	
	*qPGWC4.1*	4	20,153,796	20.1%	3.73 × 10^−7^	
	*qPGWC5.1*	5	267,911	19.8%	1.75 × 10^−6^	
	*qPGWC5.2*	5	14,313,526	20.3%	4.10 × 10^−8^	
	*qPGWC9.1*	9	12,763,755	21.1%	2.29 × 10^−6^	
	*qPGWC11.1*	11	23,382,869	18.7%	1.13 × 10^−6^	
DEC	*qDEC1.1*	1	42,431,688	16.3%	2.29 × 10^−6^	
	*qDEC6.1*	6	27,376,022	17.0%	3.19 × 10^−6^	
	*qDEC11.1*	11	7,169,791	17.3%	3.54 × 10^−6^	
	*qDEC12.1*	12	17,698,258	16.6%	2.80 × 10^−6^	

## Data Availability

Not applicable.

## Supplementary Materials

The following supporting information can be downloaded at: https://www.mdpi.com/article/10.3390/plants12030419/s1, Figure S1: Histogram of the phenotypic frequency distribution of rice grain weight, grain shape and grain chalkiness in 137 rice accessions. Table S1: Information of 137 rice accessions. Figure S2: LD decay distance estimated for 220 rice accessions. Table S2: Analysis of variance associated with grain weight, grain shape and chalkiness traits. Figure S3: Distribution of single nucleotide polymorphisms (SNPs) and nucleotide diversity across the rice Nipponbare genome in the rice association panel. Table S3: BLUP values consistently determined QTN of grain weight, grain shape and chalkiness traits by TASSEL in the three environments. Table S4: List of genes associated with grain weight, grain shape and chalkiness traits.
