# Supplementary material for: Genome-Wide Association Study of Rice Grain Shape and Chalkiness in a Worldwide Collection of Xian Accessions
Source: Plants (Basel). 2023 Jan 17;12(3):419. doi: 10.3390/plants12030419 (PMC9919668; doi:10.3390/plants12030419)
Supplement: Supplementary file 1 [file plants-12-00419-s001.zip › Supplementary Figures.pdf]

Figure S1. Histogram of the phenotypic frequency distribution of rice grain weight, grain shape and grain chalkiness in 137 rice accessions.

Figure S2. LD decay distance estimated for 220 rice accessions.

Figure S3. Distribution of single nucleotide polymorphisms (SNPs) and nucleotide diversity across the rice Nipponbare genome in the rice association panel.

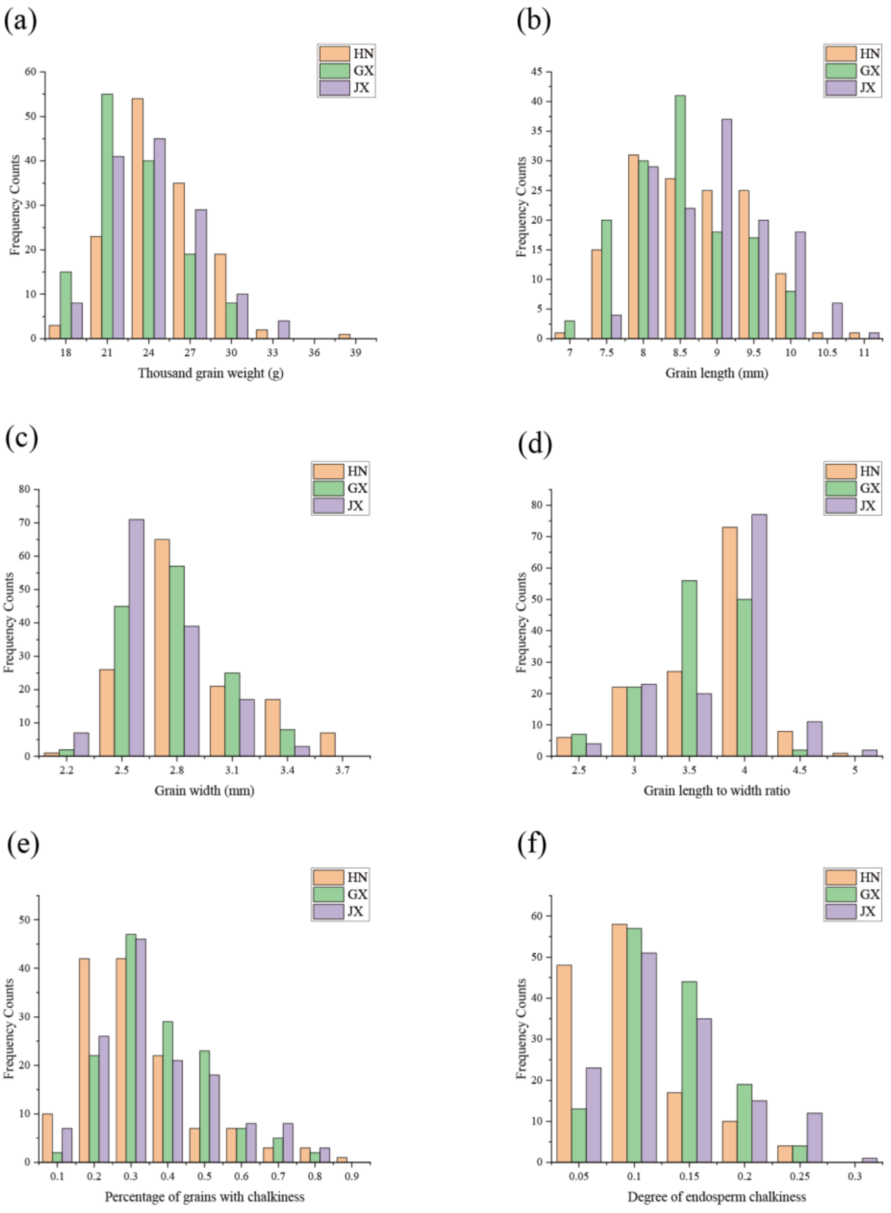

Figure S1. Histogram of the phenotypic frequency distribution of rice grain weight, grain shape and grain chalkiness in 137 rice accessions.(a) Thousand-grain weight;(b) Grain length; (c) Grain width;(d) Grain length to width ratio; (e)Percentage of grains with chalkiness; (f) Degree of endosperm with chalkiness;

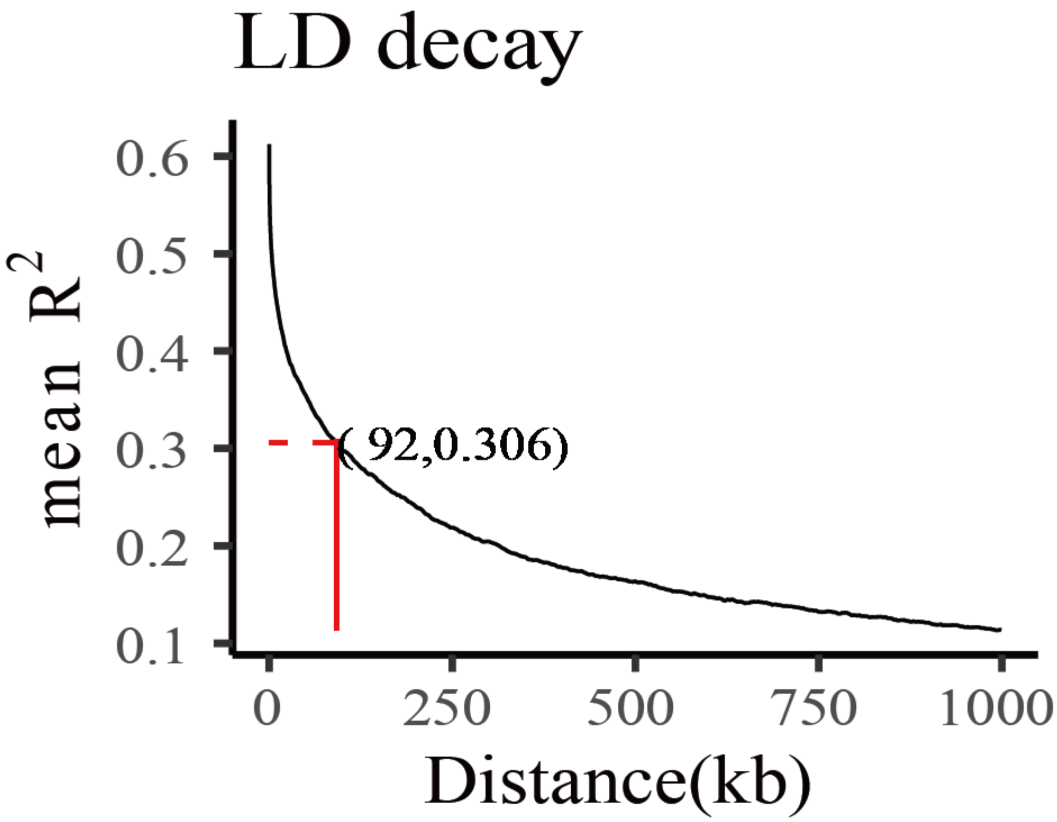

Figure S2. LD decay distance estimated for 220 rice accessions.

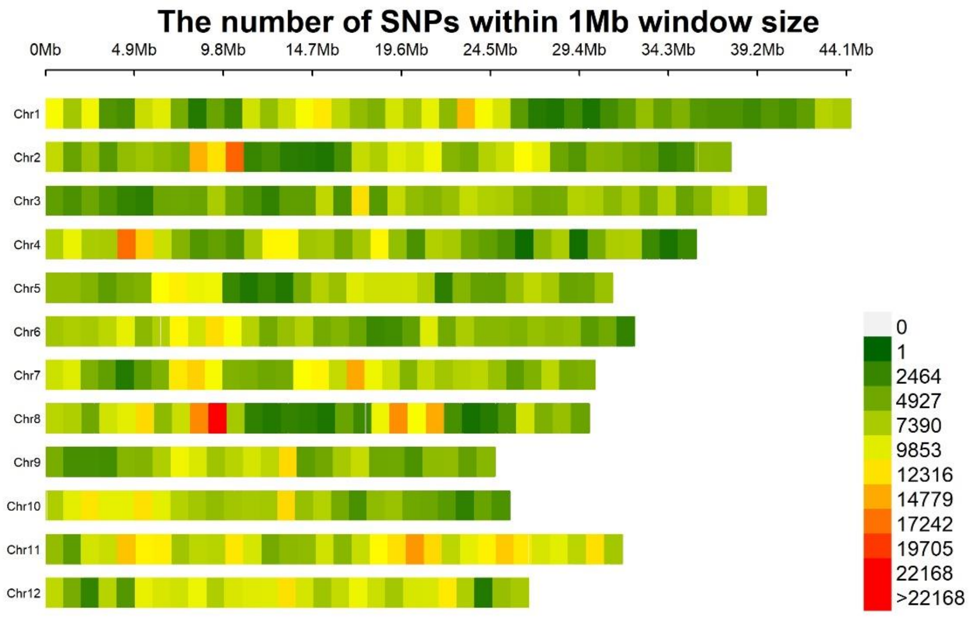

Figure S3. Distribution of single nucleotide polymorphisms (SNPs) and nucleotide diversity across the rice Nipponbare genome in the rice association panel.
